# 

**Published:** 1917-11

**Authors:** 


### THE MEDICAL BULLETIN

A Review of

### WAR MEDICINE, SURGERY AND HYGIENE

### INDEX

Volume I. November 1917 to July 1918.

### SUBJECT INDEX

ABBREVIATIONS :

R. = Research Society Report. A. = Abstract.

T. A. = Translation and Abstract. I. A. C. = Interallied Conference.

P. N. = Preliminary Note.

ABDOMEN and Thorax (Wounds of). I. A. C., 80.

ABDOMINAL Wounds. I. A. C. See also *Thorax*, 25.

ACIDOSIS. **Cannon**. R., 424.
(Observations on). **A. Prince**. R., 421.

ALKALINE, reserve (researches as to the amount of) in the blood serum of the wounded. **E. Zunz**. A., 464.

AMEBIC Dysentery. See *Dysentery*. A., 526.

AMPUTATIONS. I. A. C. The technique of amputations, particularly the treatment of nerves. **E. M. Corner**. A., 581.

ANAPHYLAXIS, rashes. **Lambert**. R., 218.

ANESTHESIA, an anatomical and experimental study of sacral. **J. Thompson**. A., 469.
By means of Stovain. **Desplas**. R., 447.
By oxygen gas and ether apparatus. **J. Gwathmey**. R., 452.
Experience in the use of nitrous oxide and oxygen with rebreathing in military surgery. **E. G. Boyle**. A., 468.
Practical methods of. **J. Gwathmey**. A., 465.

ANNOUNCEMENT. **A. Lambert**. 1.

ANTITETANUS Serum, observations with, in the monkey. **C. S. Sherrington**. A., 194.

ANTISEPTICS, flavine and brilliant green powerful, with low toxicity to the tissues : their use in the treatment of infected wounds. **C. C. Browning, R. Gulbransen, E. L. Kennaway**, and **L. H. D. Thornton**. A., 28.
Note on the germicidal power of flavine. **R. T. Hewlet**. A., 31.

ARTIFICIAL Limbs, I. A. C., 13, 76.

ASEPTIC. See *War Wounds*.

AVIATION, visual factors in equilibration. **P. Fridenberg**. A., 566.
The ear and. **I. Jones**. A., 565.
Medical aspects of flying. **J. Birley**. R., 553.

AVIATORS, the effects of high altitudes upon the efficiency of. **E. G. Seibert**. A., 562.
Some observations on the Barany tests as applied to. **H. L. Babcock**. A., 563.
The examination of. **F. A. Bachman**. A., 568.

BACTERIOLOGY, in war surgery, the value of. I. A. C., 74.
Aspect of gas gangrene. **Henry**. R., 142.
Notes on the bacteriological investigations of wounds. **L. Vaucher**. R., 286.

BLOOD Groups and the selection of donors for transfusions. **Moss**. R., 516.

BLOOD Infections. **Mullally** and **McNee**. A., 103.

BLOOD Transfusions. **W. M. Bayliss**. A., 461.

BLOOD Vessels, wounds of the. I. A. C., 18.

BONE Infection. **K. Taylor**. R., 217.

BONE, transplantation of. **W. Gallie** and **D. Robertson**. A., 580.

BRAIN Wounds. I. A. C., 80.
Gas poisoning. **F. W. Mott**. A., 572.
Examination of, in shell shock. **F. W. Mott**. A., 473.

BRILLIANT Green. See *Antiseptics*.

CEREBRAL wounds. I. A. C., 22.

CEREBRO-SPINAL Fever in the Royal Navy from 1916-17. **H. D. Hollecton**. A., 404. See also *Meningitis*.

CEREBRO-SPINAL Fever epidemic of 1917. **J. Glover**. A., 589.

CHEST, war surgery of the. **A. Lockwood** and **J. Nixon**. A., 575.
Wounds. I. A. C., 79.
Wounds penetrating the. I. A. C., 23.
Wounds, some statistical results of the treatment of. **T. Elliott**. A., 577.

CHLORINE gas, treatment of scabies. **G. Clark** and **H. Raper**. A., 164.

CLOTHIN G, the role of, in the infection of war wounds. **P. Carnot**. A., 172.

COLD, effects of, upon the body. **N. C. Lake**. A., 269.

COLON, ulceration of the, in the neighborhood of gunshot wounds. **J. S. Dunn** and **H. Drummond**. A., 40.

CRANIOPLASTY, a chondrotome for, **A. Gossett**. T. A., 39.

DEAF Soldier, treatment and training of the. **D. Grant**. A., 597.
Hysterical, the pathology, diagnosis and treatment of absolute deafness in soldiers. **A. Hurst** and **E. Peters**. A., 595.

DELOUSING. See *Louse Problem.*

DIPLOCOCCUS, occurring in skin infections, a pyogenic gram-negative. **Edward Krumbhaer**. R., 223.

DISINFECTION of wounds. I. A. C., 74.

DYSENTERY. **C. Martin**, R., 482.
Amebic. **Paul Ravaut**. R., 488.
Complications and convalescence of. **A. Howell**. A., 531.
Bacillary. **F. Rist**. R., 485.
Bacillary. **A. Ellis**. R., 486.
Bacilli, the differentiation of true, from allied species. **F. W. Andrewes**. A., 538.
The carriage of cysts of entamoeba histolytica, protozoa and eggs, by house flies. **C. M. Wenyon** and **F. W. O'Connor**. A., 526.
Carriers, detection and treatment of, with emetine bismuth iodide among cases of irritable heart. **M. Jepps** and **J. Meakins**. A., 531.
Lipovaccines, an experimental investigation of lipovaccines with a note on triple. **Withmore** and **Fennel**. A., 537.
Method of concentrating entamoeba cysts in stools. **Cooper** and **Row**. A., 525.
Notes on the etiology of. **Martin** and **Williams**. A., 532.

DYSENTERY. On the three common intestinal entamoebae of man and their differential diagnosis. **Dobell** and **Jepps**. A., 527.
Persistant carriers of entamoeba histolytica. **Shepheard** and **Lillie**. A., 537.
Reports on one thousand soldiers convalescent from diseases of the, and enteric groups. **W. Fletcher**. A., 534.
Results of the examination of the stools of 422 cases admitted to a Cairo hospital. **Martin, Kellaway** and **Williams**. A., 533.
The role of flies in the spread of. **E. Roubaud**, A., 527.
Some points in the diagnosis and treatment of, in the British Salonika Force. **D. Graham**. A., 533.
Suspect cases in France, some strains of organisms found in, showing peculiar agglutinating phenomena and sugar reactions. **F. Smartt**. A., 535.
The treatment of 102 carriers of entamoeba histolytica with emetine bismuth iodide. **Waddell, Banks, Watson**, and **King**. A., 536.

DYSENTERIAE Shiga in severe cases. **Bezancon, Ranque, Senez, Coville** and **Paraf**. A., 532.
Shiga, a case of septicemia due to infection by bacillus. **C. M. A. Maer**. A., 534.

DYSENTERIC, Amebic, peripheral neuritis following emetime treatment of. **Kilgore**. A., 526.
Anti, sero-vaccine, results obtained from the use of, in the field with regard to the reduction of case incidence **G. Gibson**. A., 524.
Ulcers, localization of, by X-Rays. **T. Holland**. A., 526.

EDITORIAL Note. 73.

EUSOL, on a convenient method of preparing. **Smith, Ritchie** and **Bettie**. A., 27.

EXTRACTING Projectiles, comparative study of the different methods of. I. A. C., 6.

FLESH Wounds in the surgical formations of the army, notes on the treatment of. **P. Duval**. R., 273.

FRACTURES. I. A. C., 14.

of the arm. I. A. C., 14.

of the ankle. I. A. C., 16.

of the fore-arm. I. A. C., 15.

of the leg. I. A. C., 15.

of the thigh. I. A. C., 15.

FRACTURES of the wrist. I. A. C., 16.
of the thigh, loop suspension in. **Heitz-Boyer** and **Pouliquen**. T. A., 395.
Thirty cases of primary suture for complicated. **Picot**. T. A., 395.
The transformation of open, into closed fractures. **Lagoutte**. T. A., 43.
Surgical treatment of. I. A. C., 16.
Surgical treatment of war. I. A. C., 76.
GANGRENE, associated with B. Oedematiens, a case of. **Dalyell**. A., 91.
General pathology of acute bacillary, arising in gunshot injuries of muscle. **Bashford**. A., 110.
Notes on the vaccination of guinea pigs with B. perfringens. **Robertson**. A., 130.
Some observations relative to, resulting from serious frost-bite. **Bertrand** and **Walther**. T. A., 35.
Gas, a case of, exhibiting unusual proofs of a blood infection. **Mullally** and **McNee**. A., 103.
Gas, as seen at the casualty stations. **Wallace**. A., 128.
Gas, as seen at the casualty clearing stations. **Wallace**. A., 106.
Gas, bacteriological aspects of. **Henry**. R., 142.
Gas, conditions of the wounds. **Taylor**. R., 144.
Gas, in limbs by the resection of infected muscles, the successful conservative treatment of early. **Frankau, Drummond** and **Nelligan**. A., 125.
Gas, in the war wounds. **Sacquépée**. T., 140.
Gas, its course and treatment. **Taylor**. A., 126.
Gas, its course and treatment. **Taylor**. A., 100.
Gas, into living muscle, the method of spread of. **McNee** and **Dunn**. A., 119.
Gas, Metastatic. **Hartley**. A., 104.
Gas, microscopic histology of. **Mc Nee**. R., 143.
Gas, some factors in the pathology of. **d'Este Emery**. A., 105.
Gas, the color changes seen in skin and muscle in. **Wallace**. A., 116.
Gas, toxin and antitoxin of, and the protective inoculation against Bacillus Welchii. **Bull** and **Pritchett**. A., 130.
GANGRENE. Gaseous, A clinical study of anaerobic wound infection. **Ivens**. A., 89.
Gaseous, antitoxic sera for. **Weinberg**. R., 145.
Gaseous, treatment. I. A. C., 75.
Gaseous, a form of, with reference to malignant gaseous edema. **Sacquépée**. T., 83.
Gaseous and hematoma. **Heitz-Boyer**. T. A., 88.
Gaseous, factors responsible for. **Taylor**. A., 85.
Gaseous, its pathological and bacteriological aspects. I. A. C.. 6.
Gaseous, studies concerning. **M. Weinberg** and **P. Seguin**. T. A., 95.
Gaseous, treatment of. I. A. C., 8.
Gaseous, treatment of, by the use of serum. **Weinberg**. A., 228.
Infections of war wounds and of gas gangrene in particular, radiographic aspects of. **Lardennois** and **Pech**. T., 113.
GAS, the first symptoms of intoxication by mustard gas. **Giraud**. T. A., 570.
Intoxication by asphyxiating, and its treatment. **Colard** and **Spehl**. T. A., 574.
Poisoning, a blood change in. **Miller**. A., 573.
Poisoning, a note on blood changes in. **Miller** and **Fainy**. A., 593.
Poisoning, punctiform hemorrhage of the brain in. **Mott**. A., 572.
Poisoning, treatment of chlorine. **A. Stuart Hebblethwaite**. 571.
HEMORRHAGES, a note on. **C. Janssen**. R., 444.
HEMOTHORAX, infection of, by anaerobic gas producing bacilli. **Elliott** and **Henry**. A., 205.
HYGIENE of the camps and cantonments of an army. **Nobécourt**. T. A., 605.
HYSTERICAL deafness, the pathology, diagnosis and treatment of absolute, in soldiers. **Hurst** and **Peters**. A., 595.
INJECTIONS, intravenous, to replace blood. **W. M. Bayliss**. A., 461.
INTERALLIED Surgical Conference. Conclusions of the Third Session (Reports). T., 74.
JAUNDICE, infective. **Bertrand Dawson**. A., 52.
LOCALIZATION, method of, and extraction of projectiles by the simultaneous use of two X-Ray tubes. **de Rio Banco**. T. A., 583.

LOUSE problem. Lieut. **Mueler**. R., 239.
The cockroach, its destruction and dispersal. **J. J. B. Holt**. A., 174.
A note on the prevention of pediculosis. **J. A. Gunn**. A., 174.
Treatment of cloth by antiseptic substances. **Mary Davies**. A., 171.
at the front. **A. D. Peacock**. A., 166.
Further observations on trench fever. **Hunt** and **McNee**., A. 170.
A method of prophylaxis against infestation with. **Rysell**. T. A., 165.
Lice and other body vermin, an investigation of the best methods of destroying. **Kinloch**. A., 169.
Lice, the use of insecticide against. **Bacot**. A., 172.
MEMBERS, artificial. **I. A. C.**, 76.
MENINGITIS along the British front. **Leishman**. R., 350.
Cerebro-spinal, diagnosis. **Bradford**. R., 352.
Cerebro-spinal. **M. Foster**. R., 346.
Cerebro-spinal. **Netter**. R., 340.
Cerebro-spinal, prevention. **Gordon**. R., 342.
Intravenous serum treatment of epidemic cerebro spinal. **Herrick**. A., 591.
Observations on serotherapy for cerebro spinal. **Dopter**. R., 338.
NERVE injuries, the so-called functional symptoms in organic. **S. B. Stopford**. A., 600.
The results of 116 operations upon the radial, because of lesions from war projectiles. **Dumas** and **Tuffier**. T. A., 207.
Wounds, treatment. **I. A. C.**, 82.
NERVES, the technique of amputations, particularly the treatment of. **Corner**. A., 581.
Preliminary reports on the pathological findings in the, following war injuries. **Cone**. A., 582.
Suture of, and alternative methods of treatment by transplantation of tendon. **Robert Jones**. R., 560.
Wounds of the. **I. A. C.**, 20.
NERVOUS functional diseases due to the war. **Schnyder**. A., 408.
NEUROSES, war. **John MacCurdy**. T. A., 470.
and psychoses, war. **Clarence Farrar**, A., 598.
The treatment of some common war. **Adrian** and **Yelland**. A., 411.
NEUROSES. War injuries and, of otological interest. **Jones-Phillipson**. A., 593.
Observations on the etiology and treatment of war. **A. F. Hurst**. A., 55.
NEPHRITIS, observations on trench, at a British hospital in France. **R. Fitz**. A., 402.
Trench, its latest stages and treatment. **M. Clarke**. A., 53.
ORTHOPEDIC surgery, the place of, in an army organization. **Joel Goldthwaite**. R., 545.
Surgery, the development of, under Colonel Sir Robert Jones. **R. Osgood**. R., 547.
Outlook, an address on the, in military surgery. **Robert Jones**. A., 556.
ORTHOPEDICS, the treatment of functional contracture by fatigue. **E. F. Reeve**. A., 559.
ORTHOPEDIST, the rôle of the, in the spontaneous foot troubles displayed by soldiers on active service. **Frohlich**. R., 551.
OSTEOMYELITIS, the treatment of the chronic, of war. **Louis Rocher**. T. A., 329.
PALUDISM, intravenous injections of quinine for the treatment of primary. **Carnot** and **de Kerdrel**. T. A., 590.
PARAPLEGIA, a new reflex sign in spastic. **Bing**. A., 601.
PLEURA, wounds of the, and of the lung by projectiles. **Roux-Berger**. T. A., 540.
PNEUMOCOCCUS types, a rapid method for the determination of. **Mitchell** and **Muns**. A., 588.
PROSTITUTION in the cantonments and in the neighborhood of the camps, its surveillance and suppression. **M. Spillmann**. T. A., 64.
PSYCHONEURÓSES, the predisposing factors of war. **J. M. Wolfsohn**. A., 406.
PYOCYANEUS infection, treatment of bacillus. **Taylor**. A., 41.
Treatment of wounds infected with bacillus. **Philip Turner**. A., 42.
REEDUCATION center, report on the military centers for soldiers evacuated from the Front. **Chevallier**. R., 549.
RESEARCH Society Reports. R., 137.
RESEARCH Society Reports on Tetanus. R., 209.
SEQUESTRA, persistence of bacteria within. **Taylor** and **Davies**. A., 398.

Scabies. **McCormac**. R., 224.
**Parkinson**. R., 226.
Treatment. **Thompson**., R., 232.
Treatment. **Darier**. R., 232.
and pediculosis. **Darier**. R., 223.
On the treatment of, and some other common skin affections in soldiers. **Adamson**. A., 161.
Over-treatment of. **Johnston**. R., 230.
Problem on active service. **MacCormac**. H., 71.
Treatment of, by sulphur vapor. **Bruce** and **Hodgson**. A., 160.
The treatment of, by chlorine gas. **Clark** and **Raper**. A., 164.
Shell Shock, examination of brains in, without visible external injury. **F. W. Mott**. A., 473.
Blood pressure and surface temperature in 110 cases of. **Edith M. N. Green**. A., 62.
Shock. **George Crile**. R., 417.
Report. **I. A. C.**, 75.
Acidemia. **A. Wright**. R., 431.
Acidosis. **Cannon**. R., 424.
Acidosis, observations on. **Alexander Prince**. R., 421.
Cases. **Hamilton Drummond**. R., 440.
The etiology and pathology of. **Roux-Berger**. R., 433.
On the early stages of wound. **E. M. Cowell**. R., 429.
Resulting from war wounds. **Vincent**. R., 428.
Traumatic. **I. A. C.**, 9.
The treatment of, by intravenous injections. **Bayliss**. A., 459.
The nature of wound. **Cannon**. A., 463.
Skin and Genito-Urinary. Reports submitted on July 15, 1917. **T. A.**, 63.
Skin and Venereal centers, concerning the best distribution of. **M. O. Pasteau**. T. A., 63.
Skull, a chondrotome for cranioplasty. **A. Gosset**. T. A., 39.
Repair of injuries to, by perforated plates. **Mitchell**. A., 36.
The reparation o cranial defects by means of cartilaginous grafts. **H. L. Warren Woodroffe**. A., 37.
Spinal Cord, complications of wounds of the. **G. Roussy**. A. 602.
Clinical aspects of hemilesions of the. 604.
Wounds of the. **I. A. C.** 21.
Spirochaetes, occurring in the urine of cases of " pyrexia of unknown origin ". **S. W. Patterson**., A., 54.
Spores, the fate of bacterial, in the animal body. **Koser** and **McClelland**. A., 401.
Surgery of War, conclusions adopted by the Interallied Surgical Conference, April 1917. **I. A. C.**, 4.
Surgery, during one year's service in the 23rd General Hospital B. E. F. France, some observations on military. **Neff** and **O'Malley**. A., 390.
Supplementary services, organization of, difficulties encountered. **Gougerot**. T. A., 67.
Sutures, indications inviting primary and secondary, of war wounds determined by bacteriological investigations. **G. Gross** and **H. Tissier**. T. A., 33.
as indicated by local temperatures, the moment for delayed primary. **H. Vignes**. T. A., 397.
Delayed primary and secondary, results of. **H. A. Ballance**. R., 286.
Notes on the bacteriological investigations of wounds, practical indications for primary. **L. Vaucher**. R., 286.
Primary, of war wounds. **Gross**, **Tissier**, **Boudard**, **Di Chiara**, and **Grimault**. T. A., 383.
of wounds in time of attack. **Marquis**, **Descazal**, **Luquet**, and **Morlot**. T. A., 388.
Primary, and antiseptic methods. **C. Wallace**. R., 362.
Primary and delayed primary, war wounds treated by. **G. E. Gask**. R., 353.
Primary and secondary, of war wounds. **M. G. Potherat**. T. A., 387.
Tendon. **Torr W. Harmer**. A., 579.
of war wounds. **Lemaitre**. 292.
of war wounds at base hospitals. **Morrison**. R., 364.
Symbiosis, bacterial, on the question of, in wound infections. **Fleming**, **Douglas** and **Colebrook**. A., 92.
Syphilis, prophylaxis of, in the war zone. **Spillman**. R., 502.
Prophylaxis. **George Thibierge**. R., 504.
Tetanus. **Foster**. A., 204.
**Penhallow**. A., 204.
Active immunization against, attempted upon man. **H. Vallee** and **L. Bazy**. T. A., 200.

TETANUS. Anaphylactic rashes. **Lambert.** R., 218.
Antitoxin, in experimental tetanus, a comparison of subcutaneous with intravenous and intrathecal administration of. **Golla.** A., 192.
Antitoxin, the intrathecal route for the administration of. **Andrewes.** A., 195.
Anti-tetanic serum, the passive immunity conferred by a prophylactic dose of. **MacConkey** and **Homer.** A., 198.
Are there "Carriers" of the B.? **Colombino.** T.A., 179.
Bacilli and bone infection. **Taylor.** R., 217.
Incidence of the disease in the British armies on the western front. **William Leishman.** R., 209.
in the British E. Force, a statistical note on an analysis of cases of. **M. Greenwood.** A., 192.
in the British E. Force, recent cases of. **Leishman** and **Smallman.** A., 187.
following serum treatment. **A. Lumière.** T.A., 199.
in home military hospitals, cases of, **Bruce.** A., 184.
The intramuscular versus the intrathecal route in the treatment of, by antitoxin. **Bruce.** A., 194.
Individual cases. **Espitalier** and **Vitoux.** A., 203.
Local. **Leishman.** R., 220.
Local. **Murphy.** R., 220.
Local and serum administration. **Cushing.** R., 219.
and massive injections of serum in the spinal column. **Pignoe, Brisset** and **Lemonnier.** T.A., 175.
Memorandum on, (War Office Committee). A., 182.
Modified. **Burrows.** A., 197.
A modern view of. **Fred Ransom.** A., 201.
and neuritis. **Mouat.** A., 203.
and neuritis. **Mullaly.** A., 204.
and neuritis. **Phelip** and **Policard.** A., 203.
Prophylaxis. **Murphy.** R., 213.
Prophylaxis and treatment. **Robertson.** R., 214.
The prophylaxis of, a summary. **Mac Conkey.** A., 176.
Recurrent. **Westwater.** A., 204.
Resulting from trench feet, prophylaxis of. **J. Bacri.** T.A., 179.
TETANUS. Statistics, an analysis of recent. **F. Golla.** A., 196.
Statistics of cases of, in the army zones. **Chavasse.** T.A., 199.
Time of administration of serum. **Thompson.** R., 222.
Treated in home hospitals, second third, fourth and fifth analyses of cases of. **Bruce.** A., 184-187.
Treatment of. (Service de Santé). T., 180.
Treatment with magnesium sulphate. **Blake.** R., 216.
Twenty five cases of. **H. R. Dean.** A., 193.
THORAX and Abdomen Wounds. I.A.C., 80.
TRANSFUSION, a simple and rapid method for the selection of suitable donors for. **Roger Lee.** A., 458.
Further observations on the results of blood, in war surgery. **Robertson** and **Watson.** A., 457.
of blood. (Lakeside unit). R., 442.
with preserved red blood cells. **O. H. Robertson.** R., 436.
with tested bloods. **Howard Kansner.** A., 455.
TREATMENT, a method of wound, by the introduction of living cultures of a spore-bearing anaerobe of the proteolytic group. **Robert Donaldson, J. Leonard Joyce.** A., 44.
TREATMENTS, study of laboratory methods for the purpose of aiding the surgeon in the choice of. I.A.C., 5.
TREATING wounds, comparison of methods of. **K. Taylor.** A., 25.
TRENCH Foot, the causes and prevention of. **Hughes.** A., 263.
The cause, prevention and cure of so-called frost bite. **Raymond** and **Parisot.** T.A., 268.
The character and treatment of frost bit. **Munroe.** A., 261.
Discussion following the report of a case of trench feet. **Quenu, Jacob, Robert, Mauclaire, Routier, Tuffier, Lenormant, Le Fort** and **Broca.** A., 265.
Frozen in the army. **Temoin.** T.A. 244.
An investigation into the effects of cold upon the body. **N. Lake.** A., 269.
A note on the after treatment of so-called "Frost-bite". **Frederick Johns.** A., 264.
Pathology of trench frost bite. **Smith, Ritchie** and **Dawson.** A., 261.

TRENCH Foot. Prevention of. *Lancet* editorial. **A.**, 259.
The treatment of frost bite and of burns. **Rathery** and **Bauzil**. **T. A.**, 267.
The treatment of frost bite by gomenol oil dressings. **Alglave. T. A.**, 246.
A study of. **Raymond. T. A.**, 255.

TRENCH Fever. **Blake. R.**, 157.
**Lee. R.**, 155.
**McNee. R.**, 146.
**Swift. R.**, 154.
**Thompson. R.**, 156.
Committee Announcement. **Blake. R.**, 159.
Investigations. **Strong, Swift, Opie, MacNeal, Baetjer, Pappenheimer** and **Peacock. R.**, 376.
A review of the present knowledge of. Editorial. **R.**, 132.

VENEREAL prophylactic ointment. **Gauducheau. R.**, 515.

VENEREAL disease, method of dealing with. **G. Walker. R.**, 497.
The social basis of the campaign against, in the A. E. F. **E. Keyes. R.**, 492.
Anti-venereal struggle outside the army zone. **Gougerot. R.**, 499.
Success of the campaign for combating, in the **A. E. F. H. Young. R.**, 505.

VENEREAL Disease in war factories, organizations of the surveillance exercised with regard to. **M. J. Nicolas. R.**, 68.

WAR Wounds, the treatment of. **F. Besley. A.**, 393.
in various stages, aseptic and physical means of treatment for. **R. Leriche. T. A.**, 123.

WOUND treatments, a method of, by the introduction of living cultures of a spore-bearing anaerobe of the proteolytic group. **R. Donaldson** and **J. L. Joyce. A.**, 44.
Study of laboratory methods for the purpose of aiding the surgeon in the choice of. **I. A. C.**, 5.
Comparison of methods of. **K. Taylor. A.**, 25.
in various stages, aseptic and physical means of treatment for. **R. Leriche. T. A.**, 123.

X-RAY examination for foreign bodies in the eye and their localization. **Belot** and **Fraudet. T. A.**, 474.
in a base hospital in France, a few observations of a practical nature. **Aylward. A.**, 587.
Simplified methods. **H. C. Gage. A.**, 45.
See *Extracting projectiles* also.

### INDEX OF AUTHORS

**Adamson**, H. G. : —
On the treatment of Common Skin Affections, A., p. 161.
**Adrian**, E. D. and **Yelland**, L. R. · —
The Treatment of Some Common War Neuroses, A., p. 411.
**Alglave**, M. and **Arrou**, M. : —
Trench Feet, Treatment of Frost-bite, A., p. 246.
**Andrewes**, F. W. : —
Intrathecal Route for the Administration of Tetanus Antitoxin, A., p. 195.
The Differentiation of True Dysentery Bacilli, A., p. 538.
**Arrou**, M. and **Alglave**, M. : —
Trench Feet, Treatment of Frost-bite, A., p. 246.
**Aylward**, B. H. S. : —
X-Ray Work in a Base Hospital, A., p. 587.
**Baetjer**, W. and **Others** : —
Report on Trench Fever Investigations, R., p. 376.
**Babcock**, H. L. : —
Some Observations upon the Barany Tests, A., p. 563.
**Bachman**, F. A. : —
Examination of Aviators, A., p. 568.
**Bacot**, A. : —
The Use of Insecticide against Lice, A., p. 172.
**Bacri**, M. J. : —
Prophylaxis of Tetanus Resulting from Trench Feet, A., p. 179.
**Ballance**, H. A. : —
Results of Delayed Primary and Secondary Suture, R., p. 372.
**Banks**, C. and **Others** : —
Treatment of 102 Carriers of Entamoeba Histolytica with Emetine Bismuth Iodide, A., p. 536.
**Bauzil**, L and **Rathery**, F. : —
Treatment of Frost-bite and Burns, A., p. 267.
**Bashford**, E. F. : —
General Pathology of Acute Bacillary Gangrene, A., p. 110.
**Bayliss**, W. M. : —
Treatment of Shock by Intravenous Injections, A., p. 459.
Intravenous Injections to Replace Blood, A., p. 461.
**Bazy**, L. and **Vallee**, H. : —
Active Immunization Against Tetanus, A., p. 200.
**Bertrand**, M. : —
Some Observations Relative to Gangrene Resulting from Serious Frost-bite, A., p. 35.
**Besley**, F. A. : —
Treatment of War Wounds. (Aseptic and Open Air Methods), A., p. 393.
**Bettie**, T. and **Others** : —
On a Convenient Method of Preparing Eusol, A., p. 27.
**Bezançon**, F. : —
B. Dysenteriae Shiga in Severe Cases, A., p. 532.
**Birley**, J. L. : —
Medical Aspects of Flying, R., p. 553.
**Blake**, J. : —
Discussion. Trench Fever, R., p. 157.
**Boudard**, L. and **Others** : —
Primary Suture of War Wounds, A., p. 383.
**Boyle**, E. G. : —
Nitrous Oxide and Oxygen with Rebreathing, A., p. 468.
**Bradford**, J. R. : —
Diagnosis of Cerebro-spinal Meningitis, R., p. 352.
**Brisset**, and **Others** : —
Tetanus and Massive Injections of Serum in the Spinal Column, T. A., p. 175.
**Broca**, and **Others** : —
Trench Feet, A., p. 265.

**Browning, C. C.** and **Others** : —
Flavine and Brilliant Green Antiseptics : Use in Treatment of Infected Wounds, **A.**, p. 28.
**Bruce, D.** : —
Cases of Tetanus Treated in the Home Hospital, **A.**, p. 184.
The Intramuscular Versus the Intrathecal Route in the Treatment of Tetanus, **A.**, p. 194.
**Bruce, J.** : —
Treatment of Scabies by Sulphur Vapor, **A.**, p. 160.
**Bull, C.** and **Pritchett, I.** : —
Toxin and Antitoxin of, and Protective Inoculation against, B. Welchii, **A.**, p. 130.
**Burrows, H.** : —
Modified Tetanus, **A.**, p. 197.
**Cannon, W. B.** : —
The Nature of Wound Shock, **A.**, p. 463.
Acidosis in Shock, **R.**, p. 424.
**Carnot, P.** : —
The Role of Clothing in Infection of War Wounds, **A.**, p. 172.
Intravenous Injections of Quinine for Paludism, **A.**, p. 590.
**Chavasse, M.** : —
Statistics of Cases of Tetanus, **A.**, p. 199.
**Chevallier** : —
Report on the Organization of Reeducational Centers, **A.**, p. 549.
**Clark, G. H.** and **Raper, H. S.** : —
The Treatment of Scabies by Chlorine Gas, **A.**, p. 164.
**Clarke, J. M.** : —
Trench Nephritis : Its Later Stages and Treatment, **A.**, p. 53.
**Colard, A.** and **Spehl, P.** : —
Intoxication by Gas and its Treatment, **A.**, p. 574.
**Colebrook, L.** and **Others** :
Studies in Wound Infections (Bacterial Symbiosis), **A.**, p. 92.
**Colombino, S.** : —
Are there Carriers of the B. Tetanus? A., p. 179.
**Cone, S. M.** : —
Pathological Findings in the Nerves, **A.**, p. 582.
**Conference** : —
Surgery of War : Conclusions adopted by the Interallied Surgical Conference, **I. A. C.**, p. 4.
**Cooper, J. W.** and **Row, F. W. H.** : —
Method of Concentrating Entamoeba Cysts in Stools, **A.**, p. 525.
**Corner, E. M.** : —
Technique of Amputations, Particularly the Treatment of Nerves, **A.**, p. 581.
**Coville**, and **Others** : —
B. Dysenteriae Shiga in Severe Cases, **A.**, p. 532.
**Cowell, E. M.** : —
Early Stages of Shock, **R.**, p. 429.
**Crile, G. W.** : —
Shock, **R.**, p. 417.
**Dalyell, E. J.** : —
A Case of Gas Gangrene Associated with B. Œdematiens, **A.**, p. 91.
**Darier, J.** : —
Louse Problem Discussion, **R.**, p. 242.
Pediculosis and Scabies, **A.**, p. 223.
Discussion on the Treatment of Scabies, **R.**, p. 232.
**Davies, M.** : —
Treatment of Cloth by Antiseptic Substances, **A.**, p. 171.
**Dawson, B.** and **Others** : —
Infective Jaundice, **A.**, p. 52.
**Dawson, J.** : —
Pathology of Trench Frost-bite, **A.**, p. 261.
**Dean, H.** : —
Twenty-five Cases of Tetanus, **A.**, p. 193.
**Descazal**, and **Others** : —
Suture in Time of Attack, **T. A.**, p. 388.
**Desplas** :
Spinal Anesthesia, **A.**, p. 447.
**Di Chiara, F.** and **Others** : —
Primary Suture of War Wounds, **A.**, p. 383.
**Dobell, C.** and **Jepps, M. W.** : —
On the Three Common Intestinal Entamoebae, **A.**, p. 527.
**Donaldson, R.** and **Joyce, J. L.** : —
A Method of Wound Treatment by Cultures of a Spore-bearing Anaerobe, **A.**, p. 44.
**Dopter** : —
Sero-Therapy (Cerebro-Spinal Meningitis), **R.**, p. 338.
**Douglas, S. R.** and **Others** : —
Studies in Wound Infections (Bacterial Symbiosis), **A.**, p. 92.
**Drummond, H.** : —
Shock Cases, **R.**, p. 440.
The Successful Conservative Treatment of Early Gas Gangrene, **A.**, p. 125.
Ulceration of the Colon in the Neighborhood of Gunshot Wounds, **A.**, p. 40.

**Dumas, R.** : —
The Results of 116 Operations upon the Radial Nerve, A., p. 207.
**Dunn, J. S.** : —
Ulceration of the Colon in the Neighborhood of Gunshot Wounds, A., p. 40.
The Method of Spread of Gas Gangrene into Living Muscle, A., p. 119.
**Duval, P.** : —
Notes on the Treatment of Flesh Wounds in the Surgical Formations of the Army, R., p. 273.
Primary Suture for Complicated Fractures, A., p. 395.
**Elliott, T. R.** : —
Some Statistical Results of Treatment of Chest Wounds, A, p. 577.
Infection of Hemothorax, A., p. 205.
**Ellis, A. W. M.** : —
Bacillary Dysentery as a War Disease, R., p. 486.
**Emery, W. D'Este** : —
Some Factors in the Pathology of Gas Gangrene, A., p. 105.
**Espitalier, and Others** : —
Tetanus, Individual Cases, A., p. 203.
**Fennel, E. A. and Whitmore, E. R.** : —
An Experimental Investigation of Lipovaccines, A., p. 537.
**Fitz, R.** : —
Observations on Trench Nephritis, P. N., p. 402.
**Fleming, A. and Others** : —
Studies in Wound Infections. (Bacterial Symbiosis), A., p. 92.
**Fletcher, W.** : —
Reports on one Thousand Soldiers Convalescent from Dysentery, A., p. 534.
**Foster, M. and Others** : —
Tetanus, A, p. 204.
Clinical Experience, R, p. 346.
**Frankau, C. H. S. and Others** : —
The Successful Conservative Treatment of Early Gas Gangrene, A., p. 125.
**Fridenberg, P.** : —
Visual Factors in Equilibration, A., p. 566.
**Frohlich** : —
The Role of the Orthopedist in Spontaneous Foot Troubles, R, p. 551.
**Gallie, W. E.** : —
The Transplantation of Bone, A., p. 580.
**Gask, G. E.** : —
Primary and Delayed Primary Suture, R., p. 353.
**Gauducheau** : —
Venereal Prophylaxis, R., p. 515.
**Gibson, H. G.** : —
Results Obtained from the Use of Anti-dysenteric Sero-vaccine, A., p. 524.
**Giraud, A.** : —
The First Symptoms of Intoxication by Mustard Gas., T. A. p. 570.
**Glover, J. A.** : —
The Cerebrospinal Fever Epidemic of 1917, A., p. 589.
**Gosset, A.** : —
A Chondrotome for Cranioplasty, A., p. 39.
**Goldthwaite J. E.** : —
The Place of Orthopedic Surgery in Army Organization, R., p. 544.
**Golla, F.** : —
A Comparison of Subcutaneous with Intravenous and Intrathecal Administration of Tetanus Antitoxin in Experimental Tetanus, A., p. 192.
An Analysis of Recent Tetanus Statistics, A., p. 196.
**Gordon** : —
Preventive Measures for Cerebro-spinal Meningitis, R., p. 342.
**Gougerot.** : —
The Anti-venereal Struggle Outside the Army Zone, R., p. 499.
**Graham, D.** : —
Diagnosis and Treatment of Dysentery in the British Salonika Force, A., p. 533.
**Green, E. M.** : —
Blood Pressure and Surface Temperature in 110 Cases of Shell Shock, A., p. 62.
**Greenwood, M.** : —
A Statistical Note on an Analysis of Tetanus, A., p. 192.
**Grimault, L. and Others** : —
Primary Suture of War Wounds, T. A., p. 383.
**Gross, G.** : — Primary Suture of War Wounds, T. A., p. 383.
Indications Inviting Primary and Secondary Sutures of War Wounds, T. A., p. 33.
**Gulbransen, R. and Others** : —
Flavine and Brillant Green Antiseptics : Use in Treatment of Infected Wounds, A., p. 28.
**Gwathmey, J. F.** : —
Anesthetic Administration and Oral Analgesia, R., p. 452.
Practical Methods of Anesthesia, A., p. 465.

**Gunn, J. A.** : —
A Note on the Prevention of Pediculosis, **A.**, p. 174.
**Harmer, T. W.** : —
Tendon Suture, **A.**, p. 579.
**Hartley J.** : —
Metastatic Gas Gangrene, **A.**, p. 104.
**Hebblethwaite, A. S.** : —
Treatment of Chlorine Gas Poisoning, **A.**, p. 571.
**Heitz-Boyer, M.** : —
Hematoma and Gaseous Gangrene, **A.**, p. 88.
Loop Suspension in Fractures of the Thigh, **A.**, p. 395.
**Henry, H.** : —
Bacteriological Aspects of Gas Gangrene, **R.**, p. 142.
Infection of Hemothorax, **A**, p. 205.
**Herrick, W. W.** : —
Intravenous Serum Treatment of Cerebro-spinal Meningitis, **A**, p. 591.
**Hewlet, R. T.** : —
Note on the Germicidal Power of Flavine, **A.**, p. 31.
**Holland, C. T.** : —
Localization of Dysenteric Ulcers by X-Rays, **A.**, p. 526.
**Hollecton, H. D.** : —
Cerebro-spinal Fever h Royal Navy, **A.**, p. 404.
**Holt,, J. J.** : —
The Cockroach. Its destruction and Dispersal, **A.**, p. 174.
**Homer, A.** : —
The Passive Immunity Conferred by a Prophylactic Dose of Anti-tetanic Serum, **A.**, p. 198.
**Howell, A.** : —
The Convalescence of Dysentery and its Complications, **A.**, p. 531.
**Hughes, B.** : —
The Causes and Prevention of Trench Foot, **A.**, p. 263.
**Hume, W. E.** : —
Infective Jaundice, **A.**, p. 52.
**Hunt, G. H.** : —
Further Observations on Trench Fever. (Lice), **A.**, p. 170.
**Hurst, A. F.** : —
Observations on the Etiology and Treatment of War Neurosis, **A.**, p. 55.
**Interallied Surgical Conference** : —
Conclusions, **I. A. C.**, p. 74.
**Ivens, M. H. F.** : —
A. Clinical Study of Anaerobic Wound Infections, **A.**, p. 89.
**Jacob** and **Others** : —
Trench Feet, **A.**, p. 265.
**Jacobs** : —
Delousing by Dry Heat Treatment, **R.**, p. 234.
**Janssen, C.** : —
Note on Hemorrhage, **R.**, p. 444.
**Jepps, M.** : —
On the Three Common Intestinal Entamoebae of Man, **A.**, p. 527.
The Detection and Treatment of Amebic Dysentery Carriers, **A.**, p. 531.
**Johns, F. A.** : —
After-Trea'ment of Frost-bite, **A.**, p. 264.
**Johnston, J. C.** : —
Discussion on Treatment of Scabies, **R.**, p. 230.
**Jones, I. H.** : —
The Ear and Aviation, **A.**, p. 565.
**Jones-Phillipson, C. E.** : —
War Injuries and Neuroses of Otological Interest, **A.**, p. 593.
**Jones R.** : — Orthopedic Outlook in Military Surgery, **A.**, p. 556.
Suture of Nerves and Transplantation of Tendon, **A.**, p. 560.
**Joyce, J. L.** and **Others** : —
A Method of Wound Treatment by Cultures of a Spore-bearing Anaerobe, **A.**, p. 44.
**Karsner, H. T.** : —
Transfusion with Tested Bloods, **A.**, p. 455.
**Kellaway** and **Others** : —
Dysentery. Results of the Examination of Stools, **A.**, p. 533.
**Kennaway, E. L.** and **Others** : —
Flavine and Brilliant Green Antiseptics : Use in Treatment of Infected Wounds, **A.**, p. 28.
**de Kerdrel, A.** : —
Intravenous Injections of Quinine for Paludism, **A.**, p. 590.
**Keyes, E. L.** : —
The Social Basis of the Campaign against Venereal Diseases in the American E. F., **R.**, p. 492.
**Kilgore** : —
Peripheral Neuritis Following Emetine Treatment of Amebic Dysentery, **A.**, p. 526.
**King, W. O. R.** and **Others** : —
Treatment of 102 Carriers of Entamoeba Histolytica with Emetine Bismuth Iodide, **A.**, p. 536.
**Kinlock, J. P.** : —
The Best Methods of Destroying Lice, **A.**, p. 169.

**Koser, S. A.** : —
Bacterial Spores in the Animal Body, A., p. 401.
**Krumbhaer, E. B.** : —
A Pyogenic Gram-Negative Diplococcus Occurring in Skin Infections, R., p. 233.
**Lagoutte, M.** : —
The Transformation of Open Fractures into Closed Fractures, A., p. 43.
**Lancet Editorial** : —
Prevention of Trench-Foot, A., p. 259.
**Lake, N. C.** : —
The Effects of Cold upon the Body, A., p. 269.
**Lakeside Unit** : —
Transfusions, R., p. 442.
**Lardennois, G.** : —
Radiographic Aspects of Gangrenous Infections of War Wounds and of Gas Gangrene in Particular, A., p. 113.
**Lee, R. I.** : —
Selection of Donors by Blood Groups, A., p. 458.
Trench Fever Discussion, R., p. 155.
**Le Fort**, and **Others** : —
Trench Feet, A., p. 266.
**Leishman, W. B.** : —
Recent Cases of Tetanus, A., p. 187.
Experience at the Front (Cerebrospinal Meningitis), R., p. 350.
Tetanus on the Western Front, R., p. 209.
**Lemaitre, R.** : —
Suture of War Wounds, p. 292.
**Lenormant**, and **Others** : —
Trench Feet, A., p. 265.
**Leriche, R.** : —
Aseptic and Physical Means of Treatment of War Wounds in Various Stages, A., p. 123.
**Lillie, D. G.** : —
Persistant Carriers of Entamoeba Histolytica, A., p. 537.
**Lockwood, A. L.** : —
War Surgery of the Chest, A., p. 575.
**Lumière, A.** : —
Tetanus Following Serum Treatment, A., p. 199.
**Luquet**, and **Others** : —
Suture in Time of Attack, A., p. 388.
**McClelland, J. B.** : —
Bacterial Spores in the Animal Body, A., p. 401.
**MacConkey, A. T.** : —
The Prophylaxis of Tetanus, A., p. 176.
The Passive Immunity Conferred by a Prophylactic Dose of Anti-tetanic Serum, A., p. 198.
**McCormac, R.** : —
Scabies, R., p. 224.
**MacCormac, H.** :
The Scabies Problem on Active Service, A., p. 71.
**MacCurdy, J. T.** : —
War Neuroses, A., p. 470.
**MacNeal, W. J.** and **Others** : —
Report on Trench Fever Investigations, R., p. 376.
**McNee, J. W.** : —
Trench Fever, R., p. 146.
Trench Fever, R., p. 157.
Microscopic Histology of Gas Gangrene, R., p. 143.
The Method of Spread of Gas Gangrene into Living Muscle, A., p. 119.
Further Observations on Trench Fever (Lice), A., p. 170.
A Case of Gangrene Exhibiting Unusual Proofs of a Blood Infection, A., p. 103.
**Maer, C. M. A.** : —
Case of Septicemia Due to Infection by Bacillus Dysenteriae Shiga, A., p. 534.
**Martin, C. J.** : —
Diagnosis of Bacillary Dysentery, R., p. 481.
Notes on the Etiology of Dysentery, A., p. 532.
Results of the Examination of Stools, A., p. 533.
**Mauclaire**, and **Others** : —
Trench Feet, A., p. 265.
**Meakins, J. C.** : —
The Detection and Treatment of Carriers in Dysentery, A., p. 531.
**Miller, J.** : —
A Note on Blood Changes in Gas Poisoning, A., p. 573.
**Mitchell, A. E.** : —
Repair of Injuries to the Skull by Perforated Plates, A., p. 36.
**Mitchel, O. W. H.** : —
A Rapid Method for the Determination of Pneumococcic Types, A., p. 588.
**Morlot** and **Others** : —
Suture in Time of Attack, A., p. 388.
**Morrison, J. T.** : —
Suture at Base Hospitals, R., p. 364.
**Moss** : —
Blood Groups and the Selection of Donors for Tranfusion, R., p. 516.
**Mott, F. W.** : —
Brain in Shell Shock, A., p. 473.
Punctiform Hemorrhages of the Brain in Gas-poisoning, A., p. 572.

**Mouat**, and **Others** : —
Tetanus Neuritis, **A.**, p. 203.
**Mullaly** : —
Tetanus Neuritis, **A.**, p. 204.
**Mullally, G. T.** : —
A Case of Gangrene Exhibiting Unusual Proofs of a Blood Infection, **A.**, p. 103.
**Mueler** : —
An Atomizer for Delousing, **R.**, p. 239.
**Munroe, H. E.** : —
Character and Treatment of Frost-bite, **A.**, p. 261.
**Muns, W. E.** : —
A Rapid Method for the Determination of Pneumococcus Types, **A.**, p. 588.
**Murphy** : —
Tetanus Prophylaxis, **R.**, p. 213.
**Neff, J.** : —
Observations on Military Surgery, **A.**, p. 390.
**Nelligan** and **Others** : —
The Successful Conservative Treatment of Early Gas Gangrene, **A.**, p. 125.
**Netter** and **Nicolle** : —
Serotherapy (Cerebro-spinal meningitis), **R.**, p. 340.
**Nicolle** and **Netter** : —
Serotherapy (Cerebro-spinal meningitis), **R.**, p. 340.
**O' Connor, F. W.** : —
The Carriage of Cysts by House Flies, **A.**, p. 526.
**O' Malley, J. G.** : —
Observations on Military Surgery, **A.**, p. 390.
**Opie, E. L.** and **Others** : —
Report on Trench Fever Investigations, **R.**, p. 376.
**Osgood, R. B.** : —
The Development of Military Orthopedic Surgery, **R.**, p. 547.
**Pappenheimer, A. M.** and **Others** : —
Report on Trench Fever Investigations, **R.**, p. 376.
**Paraf** and **Others** : —
B. Dysenteriae Shiga in Severe Cases, **A.**, p. 532.
**Parisot, J.** : —
Frost-bite, **A.**, p. 268.
**Parkinson, P.** : —
Prevention of Scabies and Pediculosis, **R.**, p. 226.
**Patterson, S. W.** : —
Spirochaetes Occurring in the Urine of Cases of Pyrexia of Unknown Origin, **A.**, p. 54.
**Peacock, A. D.** : —
The Louse Problem at the Front, **R.**, p. 166.
Report on Trench Fever Investigations, **R.**, p. 376.
**Pech** and **Lardennois** : —
Radiographic Aspects of Gangrenous Infections of War Wounds, and of Gas Gangrene in Particular, **T. A.**, p. 113.
**Penhallow** : —
Tetanus, **A.**, p. 204.
**Phelip** and **Policard** : —
Tetanus, **A.**, p. 203.
**Picot, M. G.** : —
Primary Suture foı Complicated Fractures, **T. A.**, p. 395.
**Pignol**, and **Others** : —
Tetanus and Massive Injections of Serum in the Spinal Column, **A.**, p. 175.
**Potherat, M. G.** : —
Primary and Secondary Sutures of War Wounds, **A.**, p. 387.
**Pouliquen** and **Heitz-Boyer** : —
Loop Suspension in Fractures of the Thigh. **T. A.**, p. 395.
**Prince, A. L.** ; —
Observations on Shock Acidosis, **R.**, p. 421.
**Pritchett, I. W.** : —
Toxin and Antitoxin of, and Protective Inoculation Against, Bacillus Welchii, **A.**, p. 130.
**Quenu**, and **Others** : —
Trench Feet, **A.**, p. 265.
**Ransom, F.** : —
A Modern View of Tetanus, **A.**, p. 201.
**Ranque**, and **Others** : —
B. Dysenteriae Shiga in Severe Cases, **A.**, p. 532.
**Raper, H. S.** : —
The Treatment of Scabies by Chlorine Gas, **A.**, p. 164.
**Rathery, F.** : —
Treatment of Frost-bite and Burns, **T. A.**, p. 267.
**Raymond, V.** : —
Study of Trench Feet, **T. A.**, p. 255.
Frost-bite, **A.**, p. 268.
**Ravaut, P.** : —
Amebic Infection in France during the War, **R.**, p. 488.
**Reeve, E. F.** —
Treatment of Functional Contracture by Fatigue, **A.**, p. 559.
**De Rio Branco.** : —
Method of Localizing and Extracting Projectiles by Two X-Ray Tubes, **A.**, p. 583.

**Rist**, **E**. : —
Bacillary Dysentery in France during the War, **R**., p. 485.
**Ritchie**, **J**. and **Others** : —
Pathology of Trench Frost-Bite, **A**., p. 261.
On a Convenient Method of Preparing Eusol, **A**., p. 27.
**Robert** and **Others** : —
Trench Feet, **A**., p. 265.
**Robertson**, **D**. **E**. and **Gallie** : —
The Transplantation of Bone, **A**., p. 580.
**Robertson**, **L**. **B**. : —
Results of Transfusion in War Surgery, **A**., p. 457.
**Robertson**, **M**. : —
Note on the Vaccination of Guinea Pigs with B. Perfringens, **A**., p. 130.
**Robertson**, **O**. **H**. : —
Preserved Corpuscles, **R**., p. 436.
**Robertson** : —
Tetanus Prophylaxis and Treatment, **R**., p. 214.
**Rocher**, **M**. **H**. **L**. : —
The Treatment of Chronic Osteomyelitis, **T**. **A**., p. 329.
**Roubaud**, **E**. : —
The Role of Flies in the Spread of Dysentery, **A**., p. 527.
**Routier**, and **Others** : —
Trench Feet, **A**., p. 265.
**Roux-Berger**, **J**. **L**. : —
Wounds of the Pleura and of the Lung, **A**., p. 540.
Etiology and Pathology of Shock, **R**., p. 433.
**Row**, **F**. **W**. : —
Method of Concentrating Entamoeba Cysts in Stools. **A**., p. 525.
**Rysell**, **A**. : —
A Method of Prophylaxis Against Infestation with Lice, **A**., p. 165.
**Sacquépée**, **M**. : —
A Form of Gaseous Gangrene (Malignant Gaseous Edema), **A**., p. 83.
Gas Gangrene in War Wounds, **R**., p. 140.
**Schnyder** : —
Nervous Functional Diseases Due to the War, **A**., p. 408.
**Seibert**, **E**. **G**. : —
The Effects of High Altitudes upon the Efficiency of Aviators, **A**., p. 562.
**Senez**, and **Others** : —
B. Dysenteriae Shiga in Severe Cases. **A**., p. 532.
**Seguin**, **P**. : —
Studies Concerning Gaseous Gangrene. **A**., p. 95.
**Service de Santé** : —
Treatment of Tetanus, **T**. **A**., p. 180.
**Shepheard**, **S**. : —
Persistant Carriers of Entamoeba Hystolitica. **A**., p. 537.
**Sherrington**, **C**. **S**. : —
Observations with Antitetanus Serum in the Monkey, **A**., p. 194.
**Smallman**, **A**. **B**. : —
Recent Cases of Tetanus, **A**., p. 187.
**Smartt**, **F**. **N**. **B**. : —
Some Strains of Organisms found in Dysentery Cases in France. **A**., p. 535.
**Smith**, **J**. **L**. : —
On a Convenient Method of Preparing Eusol. **A**., p. 27.
**Smith**, **L**. : —
Pathology of Trench Frost-bite. **A**., p. 261.
**Spehl**, **P**. : —
Intoxication by Gas and its Treatment, **T**. **A**., p. 574.
**Spillman**. : —
Prophylaxis of Syphilis in the War Zone, **R**., p. 502.
**Strong**, **R**. **P**. : —
A Review of the Present Knowledge of Trench Fever, **R**., p. 132.
Louse Problem, **R**., p. 240.
Trench Fever Discussion, **R**., p. 152.
Report on Trench Fever Investigations, **R**., p. 376.
**Swift**, **H**. : —
Trench Fever Discussion, **R**., p. 154.
Report on Trench Fever Investigations, **R**., p. 376.
**Taylor**, **K**. : —
Gas Gangrene. Its Course and Treatment, **A**., p. 126.
Louse Problem, **R**., p. 241.
Gas Gangrene. Its Course and Treatment, **A**., p. 100.
Factors Responsible for Gaseous Gangrene, **A**., p. 85.
Bacteria within Sequestra, **A**., p. 398.
Comparison of Methods of Treating Wounds, **A**., p. 25.
Treatment of Bacillus Pyocyaneus Infection, **A**., p. 41.
Discussion on Gas Gangrene, **R**., p. 144.
**Témoin**, **M**. : —
Frozen Feet in the Army, **A**., p. 244.
**Thibierge**, **G**. : —
Syphilis Prophylaxis, **R**., p. 504.

**Thompson, J. E.** : —
Sacral Anesthesia, **A.**, p. 469.
**Thompson** : —
Discussion Trench Fever, **R.**, p. 156.
Tetanus, **R.**, p. 222.
**Thornton, L. H.** and **Others** : —
Flavine and Brilliant Green Antiseptics : Use in Treatment of Infected Wounds, **A.**, p. 28.
**Tissier, H.** : —
Indications Inviting Primary and Secondary Sutures of War Wounds, **T. A.**, p. 33.
Primary Suture of War Wounds, **T. A.**, p. 383.
**Tuffier** and **Others** : —
Trench Feet, **A.**, p. 265.
**Turner, P.** : —
Treatment of Wounds Infected with Bacillus Pyocyaneus, **A.**, p. 42.
**Vallée, H.** : —
Active Immunization against Tetanus **A.**, p. 200.
**Vaucher, L.** : —
Notes on the Bacteriological Investigations of Wounds of the Soft Parts : Practical indications for Primary Suture, **R.**, p. 286.
**Vignes, H.** : —
The Moment for Delayed Primary Suture, **A.**, p. 397.
**Vincent, C.** : —
Shock from War Wounds, **R.**, p. 428.
**Vitoux** : —
Tetanus, **A.**, p. 203.
**Waddell** and **Others** : —
Treatment of 102 Carriers of Entamoeba Histolytica with Emetine Bismuth Iodide, **A.**, p. 536.
**Walker, G.** : —
Methods of Combating Venereal Disease in the A. E. F., **R.**, p. 497.
**Wallace, C. S.** : —
The Color Changes Seen in the Skin and Muscle in Gas Gangrene, **A.**, p. 116.
Gas Gangrene as Seen at the Casualty Clearing Stations, **A.**, p. 106 and 128.
The Clinical Aspect of Gas Gangrene, **R.**, p. 137.
Primary Suture and Antiseptic Methods, **R.**, p. 362.
**War Office Committee** : —
Memorandum on Tetanus, **A.**, p. 182.
**Watson, C. G.** : —
Results of Transfusion in War Surgery, **A.**, p. 457.
Treatment of 102 Carriers of Entamoeba Histolytica with Emetine Bismuth Iodide, **A.**, p. 536.
**Weinberg, M.** : —
Treatment of Gaseous Gangrene by the Use of Serum, **A.**, p. 128.
Studies Concerning Gaseous Gangrene, **T. A.**, p. 95.
Discussion on Gas Gangrene, **R.**, p. 144.
**Wenyon, C. M.** : —
The Carriage of Cysts by House Flies, **A.**, p. 526.
**Westwater** : —
Recurrent Tetanus, **A.**, p. 204.
**Whitmore, E. R.** : —
An Experimental Investigation of Lipovaccines, **A.**, p. 537.
**Williams, E. F.** : —
Notes on the Etiology of Dysentery, **A.**, p. 532.
**Williams, F. E.** : —
Results of the Examination of Stools, **A.**, p. 533.
**Wolfsohn, J. M.** —
Predisposing Factors of War Psychoneurosis, **A.**, p. 406.
**Woodroffe, H. L. W.** : —
The Reparation of Cranial Defects by means of Cartilaginous Grafts, **A.**, p. 37.
**Wright A.** : —
Acidosis in Shock, **R.**, p. 431.
**Yelland, L. R.** : —
The Treatment of Some Common War Neuroses, **A.**, p. 411.
**Young, H. H.** : —
Success of the Campaign for Combating Venereal Disease in the American E. F., **R.**, p. 505.
**Zunz, E.** : —
Alkaline Reserve in the Blood Serum of the Wounded, **A.**, p. 464.

